# Rare mycetoma caused by *Raghukumaria* species, a mangrove-derived dematiaceous fungus in a kidney transplant patient

**DOI:** 10.1016/j.idcr.2025.e02284

**Published:** 2025-06-10

**Authors:** Shekina Gonzalez-Ferrer, Lauren Yanagimoto-Ogawa, Ran Zhuo, Colette J. Matysiak Match, Ashley Dayo, Yuna Kang, Shangxin Yang

**Affiliations:** a Department of Pathology and Laboratory Medicine, David Geffen School of Medicine, University of California, Los Angeles, CA, USA; b Division of Infectious Diseases, David Geffen School of Medicine, University of California, Los Angeles, CA, USA

**Keywords:** Mycetoma, Dematiaceous mold, *Pleosporales*, *Raghukumaria keshaphalae*, Next-generation sequencing, Renal transplant

## Abstract

We report a rare case of mycetoma after renal transplant in a patient with distant exposure to mangroves during disaster relief efforts in the Caribbean. Histopathology and fungal culture from the skin biopsy revealed findings consistent with chronic mycetoma caused by a dematiaceous mold. Next Generation Sequencing (NGS) identified the organism as closely related to *Raghukumaria keshaphalae*, a fungus originally isolated from mangroves. This case highlights the emerging threat posed by environmental dematiaceous fungi as obscure opportunistic pathogens in the setting of natural disasters, especially for immunocompromised patients, and the pivotal diagnostic role of NGS in invasive fungal infections.

## Introduction

Dematiaceous fungi are melanized molds causing infections such as chromoblastomycosis, phaeohyphomycoses, and mycetomas [1], [2]. Humans may acquire cutaneous and subcutaneous infections from dematiaceous molds through cuts or abrasions. Dematiaceous molds can cause infections in all individuals, however those who are immunocompromised, including solid organ transplant recipients, are at higher risk to develop invasive infections [3], [4].

Mycetomas are painless, subcutaneous, tumorous masses, which classically present with draining sinuses, granules, and swelling, caused by either bacteria such as *Nocardia* (actinomycetoma) or fungi (eumycetoma). Infections are acquired by environmental exposure to contaminated soil or decaying vegetation, often from trauma with implantation of the fungi. Mycetoma is a globally occurring chronic disease, yet is endemic to tropical and subtropical regions referred to as the “mycetoma belt”, characterized by hot and dry weather with short yet heavy increments of rain [3]. Individuals with a developed mycetoma may have atypical presentations, such as lack of known trauma or with fungi not previously associated with human infection [4].

## Case

**Fig. 1:**
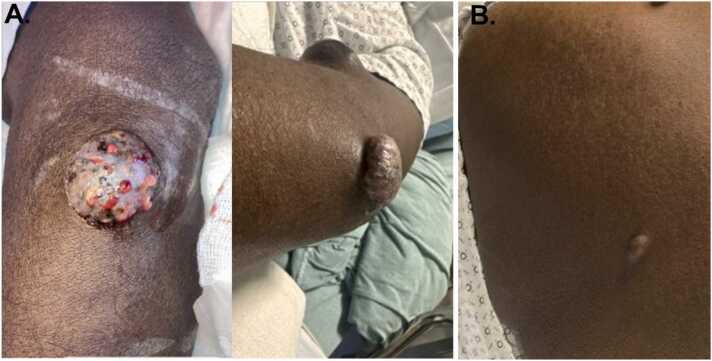
Deep cutaneous skin lesions of A) patient’s left forearm (different angles), measuring 2.5 × 1.9 cm ulnar and B) upper back.

A 50-year old male with history of deceased donor renal transplant (DDRT) in December 2023, presented to our facility in June 2024, with an enlarging skin lesion on his left forearm with biopsy concerning for fungal infection. The patient believed the lesion was present on his forearm for several months prior to DDRT but was not investigated before transplantation. The patient received anti-thymocyte globulin induction and continued maintenance immunosuppression with tacrolimus, mycophenolate mofetil, and prednisone. Post transplant the lesion gradually enlarged, and although not painful, he described it as occasionally itchy with some oozing (Fig. 1A). Within the same timeframe, he noticed a similar lesion on his left upper back, which appeared smaller yet continued to grow (Fig. 1B). He did not recall any specific injury to the area; however, he worked as a power line technician about 11 years prior, responding to natural disasters in countries throughout the Caribbean and claimed scratches and other abrasions were common on the job.

**Fig. 2:**
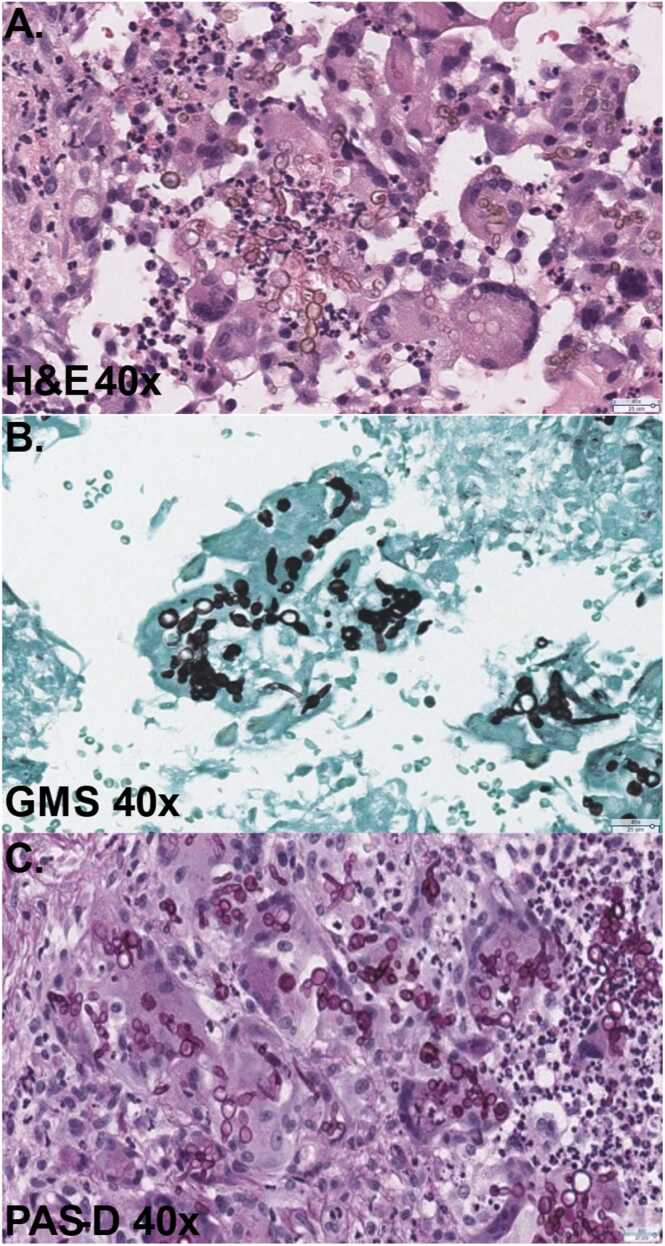
Histopathology images from a punch biopsy of the left forearm. A) H&E-stained sections (40x), showing dermal involvement by granulomatous inflammation, sclerotic bodies, with associated microabscesses with giant cells, extending to the biopsy edges. Darkly pigmented fungal hyphae are seen in both B) GMS-stained sections (40x), and C) PAS-D-stained section (40x).

A CT scan of the chest was performed which showed tree-in-bud and clustered nodules, raising concern for disseminated infection versus a separate fungal process. Histology of the skin lesion punch biopsy revealed superficial and deep dermal involvement by granulomatous inflammation, sclerotic bodies, with associated microabscesses with giant cells, extending to the biopsy edges (Fig. 2A). Hematoxylin and eosin (H&E) stained slides were consistent with chromoblastomycosis caused by dematiaceous molds, and fungal elements with pigment were identified in association with the granulomas, predominantly resembling yeast-like bodies. The GMS and PAS-D special stains highlighted the fungal formation (Fig. 2B-C).

**Fig. 3:**
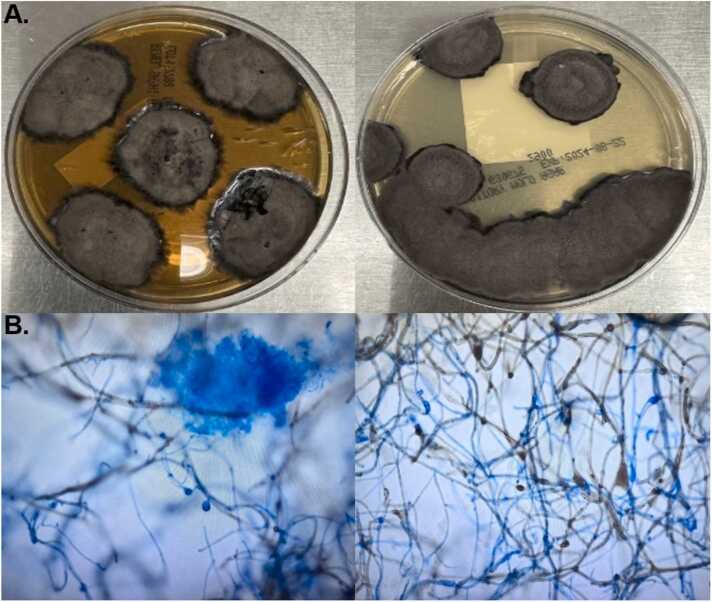
Macroscopic and microscopic fungal culture morphology. A) mold grown in routine culture media, SABHI and IMA, showing melanized colony morphology. B) microscopic images showing darkly pigmented acute angle branching, fruiting bodies, and ascospores from tape-prep of mold stained with lactophenol blue.

Fungal culture from the skin biopsy of the left forearm lesion grew a dematiaceous mold (Fig. 3A). Microscopic imaging of the mold revealed fungal hyphae with septation, acute angle branching, and dark-pigmented fruiting bodies and ascospores (Fig. 3B). Genomic identification of the mold was performed using Illumina platform based Next Generation Sequencing (NGS) as previously described [5]. The fungal isolate was sent to UT Health San Antonio Fungus Testing Laboratory for susceptibility testing, which use a broth dilution reference method for testing the susceptibility of mold according to the CLSI M38-A2.

The dematiaceous fungi isolated from the arm skin biopsy was identified as *Raghukumaria* species most closely related to *Raghukumaria keshaphalae,* order *Pleosporales* [6]. The isolate shared 96.9 % identity in its internal transcribed spacer-1 (ITS) target region with the PUFD65 strain (NCBI Accession# MK282439.1), isolated from decaying wood in India. A16S/ITS amplicon-based NGS test performed at our institution [7] also detected the same fungus directly in the Formalin-Fixed Paraffin-Embedded (FFPE) lesion tissue, as shown in Fig. 2.

Antifungal susceptibility results were shown in Table 1. While there are no proposed treatment guidelines for *Raghukumaria spp.*, the fungal lesions decreased over time and stopped draining after treatment with posaconazole 300 mg by mouth daily (MIC ≤0.03 mcg/ml), an antifungal with known successful outcomes for treatment of eumycetomas [8]. Therapeutic drug monitoring for posaconazole was performed and he has tolerated the medication without signs of toxicity. The patient eventually underwent an excision of the left forearm mass after being on posaconazole for three months. While the lesion on the left forearm significantly improved, the patient’s pulmonary nodules remained stable or slightly decreased in size, raising concern for possible other etiology. Multiple bronchoalveolar lavage (BAL) samples and biopsies have been collected and tested to determine the cause of the pulmonary nodules, but have been inconclusive. BAL cultures had no growth and histopathology exams of biopsies revealed non-necrotizing granulomatous inflammation. These changes can be seen with both infectious and non-infectious etiologies. Due to this diagnostic uncertainty and persistence of pulmonary nodules in the setting of ongoing immunosuppression, the patient will remain on posaconazole as suppression indefinitely with close monitoring.

**Table 1:** Antifungal susceptibility testing results. The fungal isolate was sent to UT health san antonio fungus testing laboratory for susceptibility testing, using a broth dilution reference according to the CLSI M38-A2.

**Antifungal**	**MIC (mcg/ml)**
5-Fluorocytosine	8
Amphotericin B	0.5
Anidulafungin	≤ 0.015
Caspofungin	≤ 0.015
Fluconazole	16
Isavuconazole	0.125
Micafungin	≤ 0.015
Posaconazole	≤ 0.03
Voriconazole	0.25

MIC: minimal inhibitory concentration.

## Discussion

This is a rare case of a human infection caused by *Raghukumaria* species which has been isolated from decaying wood of the river mangrove plant [6]. While many agents under the *Pleosporales* order have been shown to cause human mycetomas [9], [10], only a few cases have been reported in patients with a history of organ transplants [3], [4], [11], [12]. Broadly, molds in the *Pleosporales* order have been reported to cause deep cutaneous infections, presenting as sustained progressive nodular lesions, especially in immunosuppressed patients [3], [4], [12], [13]. Specifically, two cases highlighted two other marine molds under the *Trematosphaeriaceae* family*, Falciformispora lignatilis* and *Trematosphaeria grisea,* to be the causative agent of mycetoma and phaeohyphomycosis respectively [4], [12]. The first case demonstrated a renal transplant recipient sustaining a minor elbow trauma in Australia, from a lesion, caused by *Falciformispora lignatilis,* which grew over 4 months [4]. The patient migrated to Australia from the Philippines at 36 years old, thereby having ample time for inconspicuous fungal exposures in his homeland. The second case reports a heart transplant recipient with a left-hand lesion, growing *Trematosphaeria grisea,* over the course of 1-month [12]. The patient had resided in France their whole life but had taken a short trip to Tunisia 10-years prior to the presentation of the lesion. Similarly, our patient’s last exposure to mangroves was approximately 11-years ago when he was still operating as a technician in Belize. Reports have shown that lesions may gradually evolve over the course of months or even years, especially if clinically unmanaged [4], [14]. It is suspected that this fungus was introduced to the patient through traumatic inoculation while working in Belize, leading to the chronic development of fungal lesions, which were then exacerbated following renal transplantation. These three cases suggest the tropical regions influencing the seemingly unaware inoculation of these molds and long-term exposure periods leading to eventual infections.

The patient’s receipt of induction and maintenance immunosuppression for transplant, and overall increased net state of immunosuppression likely exacerbated the skin lesion, as evidenced by notable changes in size and drainage following transplantation. This is further supported by a small case series by Lum et al. where all patients also had a remote history of exposure without evidence of clinical disease until unmasked decades later after undergoing solid organ transplant [13]. Similarly, in the two prior cases caused by the *Trematosphaeriaceae* family, the transplant recipients also received induction and were on maintenance immunosuppression [4], [12].

The fungal lesion from the case presented herein did not demonstrate unique structures from fungal culture or histopathology, hence, our diagnosis heavily relied on performing NGS from the skin biopsy. This lack of definitive identification between histopathology findings and true pathogen is common amongst mycetoma-causing fungi [15]. Pathogen identification, especially in the distinguishing between eumycetoma and actinomycetoma, is important for accurate diagnosis and treatment, with NGS taking a leading role in identifying uncommon pathogens.

Our patient was successfully treated with posaconazole, which had a favorably low MIC value of ≤ 0.03 mcg/ml. Posaconazole was chosen over the more commonly used itraconazole in cases of mycetomas, as it is often better tolerated and has a broader spectrum of activity [16], [17]. Indeed, for most drugs tested (Table 1) MIC values were below 1 mcg/ml, which contrasts with several dematiaceous fungi reported to cause human infections [18]. Generally, azoles typically have demonstrated the broadest in vitro activity against dematiaceous molds, however, there are currently no randomized clinical trials to guide therapeutic decisions and duration of treatment varies based on response with consideration for surgical intervention [16]. Fluconazole uniquely had a much higher MIC value at 16 mcg/ml. This is consistent with the literature on clinical isolates of dematiaceous fungi that demonstrates higher MICs for fluconazole compared to other azoles [1], [19], [20]

## Conclusion

As climate change intensifies, leading to surges of floodwater and debris buildup, the natural reservoir for dematiaceous molds grows accordingly. An increase in natural disaster incidences will inevitably lead to more trauma related fungal infections caused by environmental fungi. Clinicians should be aware of the emerging threat posed by environmental dematiaceous fungi as obscure opportunistic pathogens in the setting of natural disasters, especially for immunocompromised patients.

## CRediT authorship contribution statement

**Shekina Gonzalez-Ferrer:** Writing – original draft, Methodology, Investigation, Formal analysis, Data curation. **Ran Zhuo:** Investigation, Data curation. **Lauren Yanagimoto-Ogawa:** Writing – original draft, Investigation, Data curation, Conceptualization. **YANG SHANGXIN:** Writing – review & editing, Supervision, Investigation, Formal analysis, Conceptualization. **Yuna Kang:** Writing – review & editing, Methodology, Investigation, Formal analysis, Data curation. **Ashley Dayo:** Methodology, Investigation, Data curation. **Colette J. Matysiak Match:** Investigation, Data curation.

## Consent

A verbal consent has been acquired from the patient.

## Ethical approval

This study does not require an IRB approval per institutional policy for a case report

## Funding

This study received no funding

## Declaration of Competing Interest

All authors declare no conflict of interest.

## Data Availability

The genome sequences generated in this study are available in the GenBank BioProject: PRJNA1237412.
